# *Streptococcus pneumoniae* nasopharyngeal carriage in Vietnamese children during the first five years of life: a post hoc analysis

**DOI:** 10.1016/j.lanwpc.2026.101805

**Published:** 2026-02-04

**Authors:** Fernanda Marincek, Beth Temple, Sam Manna, Hau Phuc Tran, Vo Thi Trang Dai, Kathryn Bright, Uyen Y. Doan, Vy Thi Tuong Le, Phuong Linh Tran, Cattram Nguyen, Thanh Van Phan, Ho Nguyen Loc Thuy, Jason Hinds, Ashleigh Wee-Hee, Leena Spry, Casey Pell, Jemima Beissbarth, Belinda Ortika, Heidi Smith-Vaughan, Huu Ngoc Tran, Thuong Vu Nguyen, Kim Mulholland, Catherine Satzke

**Affiliations:** a Infection, Immunity and Global Health, Murdoch Children's Research Institute, Melbourne, VIC, Australia; b Department of Paediatrics, The University of Melbourne, Melbourne, VIC, Australia; c Department of Microbiology and Immunology at the Peter Doherty Institute for Infection and Immunity, The University of Melbourne, Melbourne, VIC, Australia; d Pasteur Institute of Ho Chi Minh City, Ho Chi Minh City, Viet Nam; e Global and Tropical Health Division, Menzies School of Health Research, Charles Darwin University, Darwin, Northern Territory, Australia; f Child and Maternal Health Division, Menzies School of Health Research, Charles Darwin University, Darwin, Northern Territory, Australia; g Institute for Infection and Immunity, St George's, London, United Kingdom; h Department of Infectious Disease Epidemiology, London School of Hygiene & Tropical Medicine, London, United Kingdom

**Keywords:** *Streptococcus pneumoniae*, Pneumococcal carriage, Lineage, PCV, Vietnam

## Abstract

**Background:**

*Streptococcus pneumoniae* (the pneumococcus) is one of the main causes of childhood mortality. Understanding pneumococcal serotype and lineage distribution in children is important for vaccine decision-making. We undertook a secondary analysis of nasopharyngeal swabs collected from unvaccinated children as part of pneumococcal vaccine studies to provide a comprehensive picture of pneumococcal carriage epidemiology in Vietnamese children during the first 60 months of life.

**Methods:**

We analysed 4375 nasopharyngeal swabs from unvaccinated children to assess overall and vaccine-type pneumococcal carriage at 6, 12, 18, 24, and 60 months of age. For the latter three age groups, serotype distribution and genetic lineages (Global Pneumococcal Sequence Cluster, GPSCs) were described overall and by age. We also evaluated the prevalence of antimicrobial resistance (AMR) genes and multi-drug resistance (MDR), comparing vaccine-type and non-vaccine-type pneumococci.

**Findings:**

Overall pneumococcal carriage was 21·7% (952/4375) with a total of 27 serotypes detected. Serotype coverage was similar across products Pneumosil (68·6% [95% CI 65·5–71·7%], 595/867), Prevenar13 (70·0% [95% CI 67·0–73·1%], 607/867), and Vaxneuvance (70·0% [95% CI 67·0–73·1%], 607/867), and lower for Synflorix (41·5% [95% CI 38·2–44·8%], 360/867) p < 0·05 vs Pneumosil, Prevenar13 or Vaxneuvance. In total, 2444 swabs were tested at 18, 24, and 60 months. Thirty distinct GPSCs were identified, with their distribution remaining stable across these ages. AMR genes were highly prevalent, detected in 98·9% (360/364) of samples.

**Interpretation:**

Synflorix provided lower serotype coverage than other PCVs, largely driven by the prevalence of serotype 6A, which is not included in Synflorix formulation. Serotype, lineage distribution, and prevalence of AMR genes across the sampled age groups remained consistent, indicating that these distributions are broadly representative of young unvaccinated children, helping to guide optimal approaches for pneumococcal surveillance in low- and middle-income countries.

**Funding:**

10.13039/501100000925National Health and Medical Research Council, 10.13039/100000865Bill & Melinda Gates Foundation, 10.13039/100014555Murdoch Children's Research Institute.

**Research in context**

**Evidence before this study**

The introduction of pneumococcal conjugate vaccines (PCVs) has substantially reduced the burden of pneumococcal disease in many countries. However, the pneumococcus remains a leading cause of morbidity and mortality in young children globally. As a result of widespread vaccine introduction, most research has focused on vaccinated populations, so there are limited data on pneumococcal carriage in children from unvaccinated settings. We assessed the existing evidence on pneumococcal carriage from low- and middle-income countries (LMICs) in the Western Pacific region through a PubMed search (without language restrictions) for studies published between 2000 and 2025 including search terms: “*Streptococcus pneumoniae*”, “pneumo∗”, “colon∗”, “carriage”, and “serotype”. Our search revealed 14 studies including children older than 24 months in LMICs. Antimicrobial resistance (AMR) is a critical global health issue but remains poorly characterised in the region. We identified only 13 studies from the Western Pacific region that have examined AMR in carriage. These investigations primarily focus on a limited number of antibiotics and rarely explored the prevalence of specific AMR genes. In addition, 16 studies in the region used molecular epidemiological methods. Specifically, only two studies from Vietnam used multilocus sequence typing (MLST) to characterise pneumococcal genotypes, and none reported data on Global Pneumococcal Sequence Clusters (GPSCs).

**Added value of this study**

This study provides important new data on pneumococcal carriage in unvaccinated children in a LMIC setting in the Western Pacific region. We found that majority of serotypes carried are vaccine-type (Pneumosil-type, Prevenar13-type or Vaxneuvance-type) for all children under five years of age, highlighting the potential benefits of introducing any of these vaccines in Vietnam. We found that the prevalence of AMR genes and multi-drug resistance (MDR) was higher in vaccine-types compared with non-vaccine-types, a pattern that was consistent across all ages sampled. In addition, this study is the first to characterise pneumococcal lineages (GPSCs) in Vietnam, providing valuable baseline data for future vaccine impact assessments and contributing to a more comprehensive representation of genomic data from the Western Pacific region. Given the evolving landscape of pneumococcal vaccination, with shifts toward reduced-dose schedules and higher-valency vaccines that are associated with lower immunogenicity, monitoring the effectiveness of pneumococcal vaccination programs will remain critical. We found that carriage data from children sampled at any age between 6 and 60 months can reliably represent the pneumococcal population in early childhood, informing practical solutions for surveillance in resource-limited settings.

**Implications of all the available evidence**

Overall, the evidence indicates that the serotypes and lineages circulating in Vietnam are similar to those present more widely across the Western Pacific, demonstrating that data from Vietnam are relevant more broadly. Moreover, our data from an unvaccinated population informs vaccine strategies and provides a baseline for future vaccine impact studies across the region.

## Introduction

*Streptococcus pneumoniae* (the pneumococcus) is a leading cause of morbidity and mortality in young children.^1^ Pneumococci commonly colonise the nasopharynx, which is referred to as carriage. Carriage is considered a pre-requisite for the development of pneumococcal diseases such as pneumonia, meningitis or bacteraemia.^1^ The polysaccharide capsule is a key virulence factor.^1^ Serotypes are distinguished based on antigenic differences in the capsule structure; more than 100 serotypes have been recognised.^2^^,^^3^

Pneumococcal Conjugate Vaccines (PCVs) provide protection against pneumococcal disease caused by vaccine serotypes. Serotype replacement, where the prevalence of non-vaccine serotypes increase in carriage and disease, is a common phenomenon following vaccine introduction.^4^ As of 2025, PCVs are part of the infant immunisation schedule in 171 countries worldwide.^5^ However, some low- and middle-income countries (LMICs) face challenges and are yet to introduce pneumococcal vaccination into their National Immunisation Programs, with the major barrier being the high cost of the vaccines.^6^ In Vietnam, pneumonia is the primary reason for hospitalisation among children, accounting for a significant burden on the healthcare system.^7^ PCVs are currently only available in the private market, with plans to incorporate PCV into the routine vaccination schedule from 2025.^8^

Given that Asia–Pacific has a high burden of pneumococcal disease, a deeper understanding of pneumococcal microbiology in this region is crucial. Most studies in the region have been conducted in post-PCV introduction settings, meaning there are few data from unvaccinated populations. Additionally, information on pneumococcal carriage beyond 24 months is scarce despite its importance, with toddlers and preschoolers significantly contributing to community transmission.^9^

Previously, we led a series of randomised control trials in Ho Chi Minh City, Vietnam—the Vietnam Pneumococcal Program—including two main trials, the Vietnam Pneumococcal Trial I (VPTI),^10^ and the Vietnam Pneumococcal Trial II (VPTII),^10^^,^^11^ as well as a follow-up study (VPTII-b^12^). Here, we utilised nasopharyngeal swabs from unvaccinated children from these studies to provide a comprehensive picture of pneumococcal microbiology and epidemiology in Vietnamese children during the first 60 months of life. These data shed light on serotypes, antimicrobial resistance, and genetic diversity of pneumococci in Vietnam, and the potential impact of future vaccine implementation.

## Methods

A total of 4375 swabs were included: 918 from the VPTI trial, 3091 from the VPTII trial, and 366 from the VPTII-b study.

### Study site, design, participants and swab collection

VPTI, VPTII, and VPTII-b were carried out in Districts 4, 7, and 8 of Ho Chi Minh City. The design, inclusion and exclusion criteria for VPTI and VPTII have been published previously.^10^^,^^11^ In brief, these studies enrolled healthy children from the community with no significant medical or perinatal history. A summary of the study protocols, nasopharyngeal swab collection, laboratory procedures and ethical approval is provided in Supplementary Methods S1.1 to S1.4. For the current study, we included all children aged 6–60 months that were participants in any of three previous studies (VPTI, VPTII, and VPTII-b) who had not received any doses of PCV at the time of swab collection. This study includes nasopharyngeal swabs collected at the same ages across VPTI and VPTII: 6 months (n = 982), 12 months (n = 949), 18 months (n = 1150), and 24 months (n = 928), with additional swabs collected at 60 months (n = 366) from the VPTII-b.

### Serotyping

Details for serotyping procedures are described in Supplementary Method S1.3. In brief, swabs collected at 6 and 12 months were screened for pneumococcus using traditional culture by plating on horse blood agar plates containing 5 μg/ml gentamicin followed by identification including colony morphology and optochin susceptibility, followed by serotyping by latex agglutination/Quellung.^13^^,^^14^ Swabs collected at 18, 24, and 60 months were screened for pneumococcus using quantitative polymerase chain reaction (PCR) targeting the *lytA* gene.^15^ Samples that were positive or equivocal were cultured on horse blood agar plates supplemented with 5 μg/ml gentamicin and serotyped by DNA microarray (Senti-SP version 1.5, BUGS Bioscience) as described previously.^16^ Serotypes from swabs with multiple serotype carriage were classified as either major serotype (the serotype with the highest percent abundance in each sample) or minor serotypes (the other serotypes present in lower abundance within the same samples).

### AMR analysis

We compared the detection of AMR genes by DNA microarray between vaccine-type and non-vaccine-type for the products Synflorix (PCV10-GSK), Pneumosil (PCV10-SIIL), Prevenar13 (PCV13), and Vaxneuvance (PCV15) at 18-, 24-, and 60-months using Fisher's Exact test. Multi-drug resistance (MDR) was defined as pneumococci with three or more AMR genes detected. The analysis was restricted to samples containing a single pneumococcal type with no other species present as previously described.^16^

### Lineage analysis

Analyses of genetic lineages (defined by Global Pneumococcal Sequence Cluster (GPSC)), was conducted as previously described.^17^ In brief, GPSCs were inferred for the major serotypes from DNA microarray data. Samples that had predominantly non-pneumococci detected were excluded from further analysis. The proportion of samples belonging to each GPSC was plotted using R package ‘ggplot2’ (version 3.3.5).

### Statistical analysis

Statistical analyses were conducted using Stata (version 17.0) or GraphPad Prism (version 8.0.2). Nasopharyngeal samples that were *lytA* qPCR positive (Ct < 35) but could not be serotyped (either culture negative or low DNA yield from DNA extraction) were classified as pneumococcal positive with an unknown serotype and included in the overall carriage prevalence analysis. Multiple serotype carriage was assessed both including and excluding non-typeable strains. Prevalence at each age was determined by dividing the number of carriers by the total number of participants with available microbiology results, presented as a percentage with 95% confidence interval (CI). Vaccine coverage was calculated as the proportion of isolates of a vaccine serotype out of the total number of capsular pneumococci detected. The serotypes included in each vaccine were as follows: Synflorix (serotypes 1, 4, 5, 6B, 7F, 9V, 14, 18C, 19F, 23F), Pneumosil (serotypes 1, 5, 6A, 6B, 7F, 9V, 14, 19A, 19F and 23F), Prevenar13 (includes Synflorix serotypes plus 3, 6A, 19A), and Vaxneuvance (includes Prevenar13 serotypes plus serotypes 22F, and 33F). Bacterial density data were log_10_ transformed and reported as median log_10_ genome equivalents/ml (log_10_ GE/ml) with interquartile range (IQR). Continuous variables were reported as median and IQRs, and Mann–Whitney Test was used to compare groups. Categorical data were presented as counts and percentages and differences were assessed using Fisher's Exact test. All p-values were two-tailed, and a p-value of ≤0·05 was considered statistically significant.

### Ethics approval

VPTI received ethical approval from the Human Research Ethics Committee of the Northern Territory Department of Health and Menzies School of Health Research (EC00153) and the Vietnam Ministry of Health Ethics Committee. VPTII received ethical approval from the Human Research Ethics Committee of the Royal Children's Hospital Melbourne (HREC36027) and the Vietnam Ministry of Health Ethics Committee. VPTII-b received ethical approval from the Human Research Ethics Committee of the Royal Children's Hospital Melbourne (HREC85642) and the Pasteur Institute of Ho Chi Minh City Institutional Review Board. VPTI and VPTII are both registered at ClinicalTrials.gov (NCT01953510 and NCT03098628, respectively).

### Role of funding source

The VPTI trial was supported by the National Health and Medical Research Council of Australia (grant number 566792) and the Gates Foundation (grant number OPP1116833); VPTII and VPTII-b work was supported by the Gates Foundation (grant number: INV-004916). The Murdoch Children's Research Institute is supported by the Victorian Government's Operational Infrastructure Support Program. The funders of the study had no role in study design, data collection, data analysis, data interpretation, or writing of the report. The corresponding author had full access to all the data in the study and had final responsibility for the decision to submit for publication.

## Results

Overall, 4375 swabs were collected from 3124 unvaccinated children aged 6–60 months.^10^^,^^11^ Similar pneumococcal carriage prevalences were observed between VPTI and VPTII at all shared time points (Supplementary Table S1). Participant characteristics were also comparable across the two studies, with the only difference being a 0·2 month difference in median age between studies for the 18-month swab (Supplementary Table S2). As this age difference is unlikely to have any meaningful effect on carriage parameters, data from VPTI and VPTII were combined for subsequent analyses.

Overall pneumococcal carriage across all ages was 21·7% (952/4375); 15·8% (155/982) at 6 months, 22·9% (217/949) at 12 months, 25·7% (296/1150) at 18 months, 24·6% (228/928) at 24 months, and 15·3% (56/366) at 60 months (Table 1). Pneumococcal capsular carriage across all ages was 18·7% (818/4375), with most (773/818, 94·5%) pneumococcal-positive samples containing a single serotype. Multiple capsular pneumococcal carriage among positive pneumococcal samples ranged from 0·9% (2/217; 95% CI 0·1–3·2) at 12 months to 6·6% at 24 months (15/228; 95% CI 3·7–10·6) as shown in Supplementary Table S3. Including non-encapsulated pneumococci, multiple serotype carriage was still low at 7·7% (73/953).

**Table 1:** Serotype proportion (n/N) and carriage prevalence % (95% CI) in current and upcoming PCV vaccines in unvaccinated children at 6, 12, 18, 24, and 60 months of age in Vietnam.

	6m	12m	18m	24m	60m
Any pneumococcus	155/982	217/950	296/1150	228/928	56/366
	15·8 (13·5–18·2)	22·9 (20·2–25·7)	25·7 (23·2–28·4)	24·6 (21·8–27·4)	15·3 (11·7–19·4)
Synflorix-type	55/982	81/950	123/1150	87/928	14/366
	5·6 (4·2–7·2)	8·5 (6·8–10·5)	10·7 (8·9–12·6)	9·4 (7·6–11·4)	3·8 (2·1–6·4)
Prevenar13-type	93/982	133/950	196/1150	131/928	29/366
	9·5 (7·7–11·5)	14·0 (11·9–16·4)	17·0 (14·9–19·3)	14·1 (11·9–16·5)	8·0 (5·4–11·2)
Pneumosil-type	91/982	129/950	191/1150	130/928	29/366
	9·3 (7·5–11·2)	13·6 (11·5–15·9)	16·6 (14·5–18·9)	14·0 (11·8–16·4)	7·9 (5·3–11·1)
Vaxneuvance-type	93/982	133/950	196/1150	131/928	29/366
	9·5 (7·7–11·5)	14·0 (11·9–16·4)	17·0 (14·9–19·3)	14·1 (11·9–16·5)	7·9 (5·3–11·1)

n/N represents the number of positive samples containing the specified vaccine type (n) out of the total pneumococcus-positive cases (N) at that age.

PCV10-GSK (Synflorix) serotypes: 1, 4, 5, 6B, 7F, 9V, 14, 18C, 19F, 23F.

PCV13 (Prevenar13) serotypes: PCV10-GSK serotypes plus serotypes 3, 6A, and 19A.

PCV10-SIIL (Pneumosil) serotypes: 1, 5, 6A, 6B, 7F, 9V, 14, 19A, 19F, and 23F.

PCV15 (Vaxneuvance) serotypes: include PCV13 serotypes plus serotypes 22F, and 33.

**Fig. 1:**
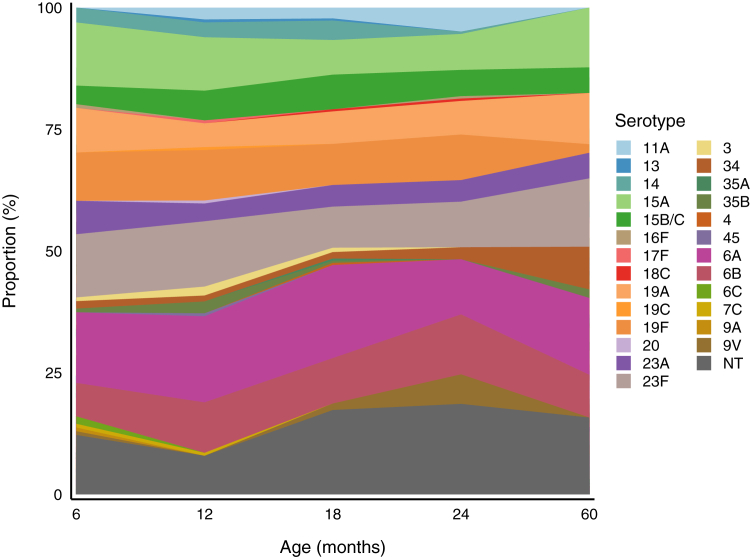
**Proportion of each pneumococcal serotype (%) among all pneumococcal-positive swabs at 6 months (n = 158), 12 months (n = 223), 18 months (n = 337), 24 months (n = 254), and 60 months (n = 57) in unvaccinated Vietnamese children. NT, non-typeable pneumococci**.

A total of 1029 pneumococci were detected (n = 867 capsular pneumococci; n = 162 non-typeable pneumococci), representing 27 different serotypes. The most common serotypes were 6A (158/1029, 15·3%), 6B (114/1029, 11·1%), 23F (111/1029, 10·8%), 19F (102/1029, 9·9%), 15A (84/1029, 8·2%), 19A (71/1029, 6·9%), 15B/C (57/1029, 5·5%), and non-typeable (162/1029, 15·7%); together these accounted for 83·5% (859/1029) of all the pneumococci detected. The relative proportion of each serotype was similar across the ages examined (Fig. 1). In the 73 samples with multiple serotype carriage there were 73 major serotypes and 77 minor serotypes (n = 69 with two serotypes, and n = 4 with three serotypes). The most common major serotypes were 23F (11/73, 15·1%), 6A (10.73, 13·7%), 19F (9/73, 12·3%), and non-typeable (12/73, 16·4%), the most common minor serotypes were 6A (11/77, 14·3%), 19F (10/77, 13%), and non-typeable (31/77, 40·2%), as shown in Supplementary Table S4.

We examined the vaccine-type carriage prevalence for the serotypes included in current available paediatric PCVs, specifically: 10 valent (Synflorix and Pneumosil), 13 valent (Prevenar13), and 15 valent (Vaxneuvance) vaccines (Table 1). Serotype coverage was similar across Pneumosil, Prevenar13 and Vaxneuvance, despite the differences in valency between products. Pneumosil-type carriage (191/1150, 16·6%) was higher than Synflorix-type carriage (123/1150, 10·7%) despite both being 10-valent vaccines, driven by the high prevalence of serotype 6A and 19A (included in Pneumosil), and low prevalence of serotypes 18C and 4 (included in Synflorix).

**Fig. 2:**
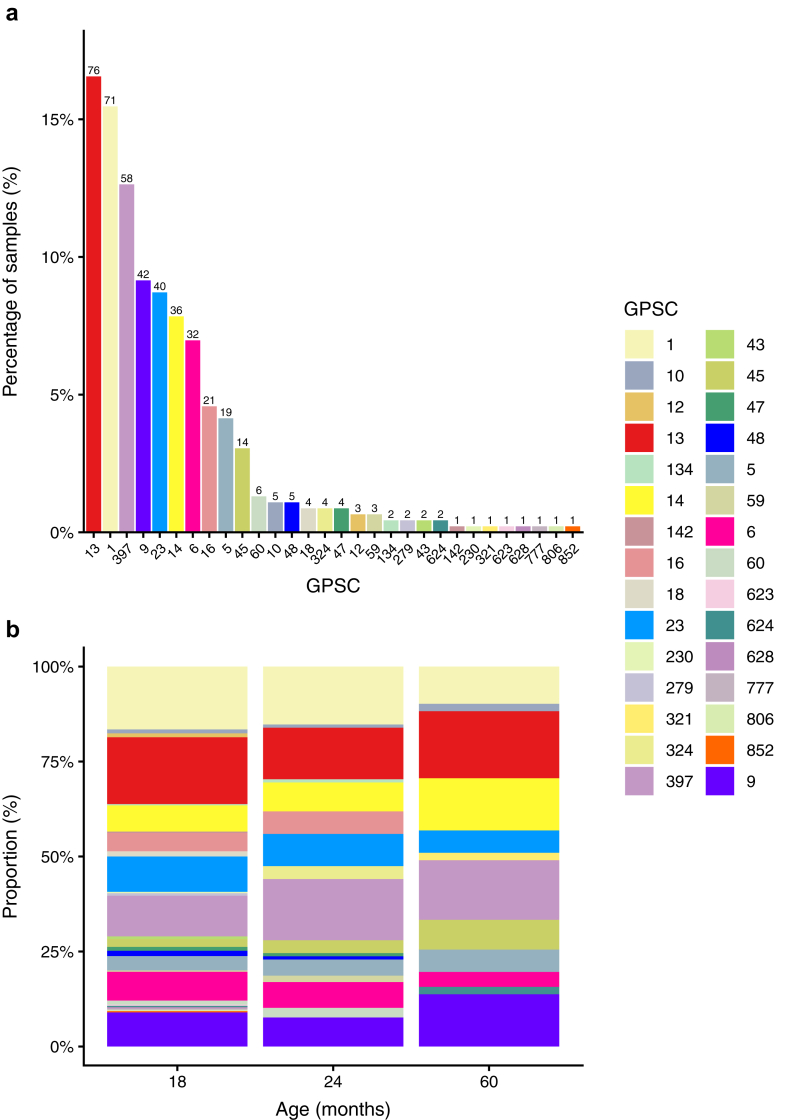
**Proportion of pneumococcal positive samples belonging to different GPSC lineages. Lineage data are inferred from DNA microarray results; therefore, ages 6 and 12 months are not included. Panel a shows the overall lineage prevalence across all included ages (18, 24 and 60 months), as a percentage. Numbers above the bars indicate the total number of pneumococcal samples identified for each GPSC. Panel b shows the relative distribution of GPSCs at 18 months (n =295), 24 months (n = 118) and 60 months old (n = 51). GPSCS were inferred for the calls with the highest relative abundance using DNA microarray. GPSC, global pneumococcal sequence cluster**.

To examine the lineage composition of pneumococci, we assessed genetic lineage from the 513 swabs with DNA microarray data available. Of these, lineage could be inferred from 464 samples (90·4%); 295, 118 and 51 samples from children aged 18 months, 24 months, and 60 months, respectively. The remaining 49 samples were excluded because non-pneumococcal species were detected at the highest relative abundance which confounds GPSC assignment. In total, 30 GPSCs were identified across all ages. GPSC13, GPSC1, GPSC397, GPSC9, GPSC23, GPSC14, GPSC6, GPSC16, GPSC5 and GPSC45 are the most common GPSCs in this population which together accounted for 89·1% of all pneumococci examined (Fig. 2). When stratified by age, the majority of GPSCs were identified across all groups, and their relative proportions remained broadly similar across ages, with some variation at 60 months (Fig. 2).

When examined by serotype, we observed that serotypes 19F, 11A, 3, and 34 were each comprised of one lineage (GPSC1, GPSC6, GPSC12, GPSC45, respectively). In contrast, other serotypes comprised of multiple GPSCs, including serotypes 14 (GPSC9, GPSC8, GPSC1, GPSC279, and GPSC6), 15B/C (GPSC6, GPSC16, and GPSC48), 23A (GPSC5, GPSC10 and GPSC6) 23F (GPSC14, GPSC16, GPSC624, and GPSC9), and 6B (GPSC23, GPSC13, GPSC47, GPSC321, and GPSC852). Of note, GPSC397 was found exclusively in non-encapsulated pneumococci, as detailed in Supplementary Table S5. For the six most prevalent serotypes in this population, the distribution of lineages within each serotype generally remained consistent across the ages examined. Exceptions included serotype 23F, where GPSC16 was not detected; 15B/C, where GPSC16 and GPSC48 were absent; and 6B, where GPSC321 emerged while GPSC852 and GPSC47 were not detected at 60 months, as represented in Supplementary Figure S1.

Data on the presence of 10 AMR genes were analysed from samples with DNA microarray data that contained a single pneumococcal serotype with no other species present (n = 364). The presence of AMR genes was very common across all ages, with at least one AMR gene found in 98·3% (230/234), 100% (89/89), and 100% (41/41) of the samples at 18, 24, and 60 months, respectively. At all ages, the most prevalent AMR genes were *ermB* and *tetM*, conferring resistance to erythromycin and tetracycline, respectively.

We examined AMR prevalence in vaccine-types and non-vaccine-types for the current available paediatric PCVs, specifically 13 valent (Prevenar13) vaccine (Table 2) as well as 10 valent (Synflorix and Pneumosil), and 15 valent (Vaxneuvance) vaccines (Supplementary Tables S6–S8). Two AMR genes (*cat* and *mefA,* conferring resistance to chloramphenicol and macrolides, respectively) exhibited a higher prevalence in Prevenar13-types compared with non-Prevenar13-types (Table 2). The *cat* gene was detected in a higher proportion of Prevenar13-types at 18 months (P < 0·001) and at 24 months (P = 0·001) compared with non-Prevenar13-types. Similarly, the *mefA* gene, was detected in a higher proportion of Prevenar13-types at 18 months (P < 0·001) and at 24 months (P = 0·005) compared with non-Prevenar13-types. We observed that MDR was higher in Prevenar13-types compared with non-Prevenar13-types at 18 and 24 months (P < 0·001) (Table 2). Similar trends were observed when comparing AMR prevalence in Synflorix-type versus non-Synflorix-type; Pneumosil-type to non-Pneumosil-type, at 18, 24 and 60 months (Supplementary tables S6–S8). The proportion of AMR among Vaxneuvance-types is the same as for Prevenar13-types, given the additional serotypes in Vaxneuvance (22F and 33F) were not detected.

**Table 2:** AMR in unvaccinated Vietnamese children aged 18, 24, and 60 months as detected by DNA microarray. The prevalence of AMR genes in samples containing Prevenar13-serotypes and non-Prevenar13-serotypes is reported.

AMR gene	18 months			24 months			60 months		
	Prevenar13-type (N = 147)	Non-Prevenar13-type (N = 55)	P value^a^	Prevenar13-type (N = 37)	Non-Prevenar13-type (N = 30)	P value^a^	Prevenar13-type (N = 24)	Non-Prevenar13-type (N = 14)	P value^a^
*aphA3*	3 (2%)	0 (0%)	0·383	3 (8%)	1 (3%)	0·390	–	–	–
*cat*	47 (32%)	3 (5%)	<0·001	18 (49%)	3 (10%)	0·001	5 (23%)	2 (14%)	0·433
*ermB*	132 (90%)	52 (94%)	0·223	37 (100%)	29 (97%)	0·448	20 (91%)	14 (100%)	0·367
*ermC*	9 (6%)	3 (5%)	0·579	6 (16%)	3 (10%)	0·355	2 (9%)	0 (0%)	0·367
*mefA*	50 (34%)	3 (5%)	<0·001	11 (30%)	1 (3%)	0·005	6 (27%)	0 (0%)	0·038
*tetK*	5 (3%)	2 (4%)	0·613	3 (8%)	1 (3%)	0·390	0 (0%)	1 (7%)	0·389
*tetM*	135 (92%)	53 (96%)	0·212	37 (100%)	29 (97%)	0·448	20 (91%)	14 (100%)	0·367
*tetO*	0 (0%)	0 (0%)	–	0 (0%)	1 (3%)	0·448	0 (0%)	0 (0%)	–
*tetL*	0 (0%)	0 (0%)	–	0 (0%)	0 (0%)	–	0 (0%)	0 (0%)	–
*sat4*	3 (2%)	0 (0%)	0·383	3 (8%)	0 (0%)	0·162	0 (0%)	0 (0%)	–
any AMR gene	144 (98%)	54 (98%)	0·701	37 (100%)	30 (100%)	–	22 (100%)	14 (100%)	–
Multi-drug resistance^b^	91 (62%)	5 (12%)	<0·001	30 (81%)	10 (29%)	<0·001	11 (50%)	3 (21%)	0·085

AMR, Antimicrobial resistance genes.

AMR and their associated resistance: *aphA3* (kanamycin), *cat* (chloramphenicol), *ermB, ermC* (erythromycin), *mefA* (macrolides), *tetK, tetM, tetO, tetL* (tetracycline), *sat4* (streptothricin). The analysis was restricted to samples containing a single pneumococcal type with no other species present. Dashes (−) indicate cases where statistical testing was not performed due to the absence of the respective AMR genes in both groups, or in cases where both groups were identical.

a The difference between Prevenar13-types vs non-Prevenar13-types was calculated using Fisher's Exact test.

b Multi-drug resistance is defined as the presence of three or more AMR genes.

**Fig. 3:**
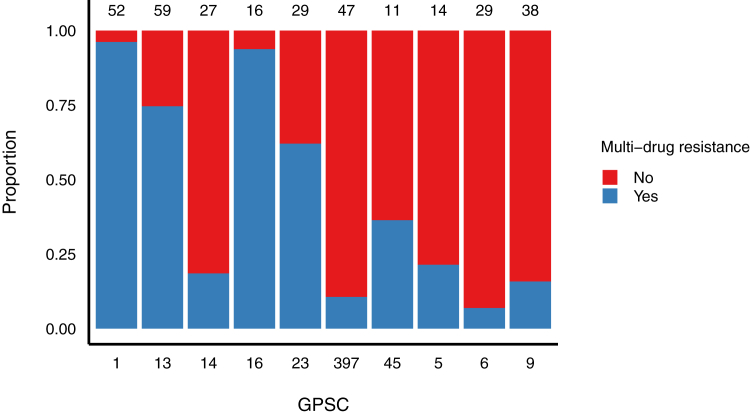
**Proportion of multi-drug resistance isolates (MDR) among Global Pneumococcal Sequence Clusters (GPSCs). Bars are scaled from 0 to 1, showing the relative proportion of MDR and non-MDR isolates within each GPSC. The total number of samples for each GPSC is displayed on the top of the corresponding bar. MDR was defined as the presence of three or more antimicrobial resistance genes. Analysis was restricted to samples with a single serotype and only GPSCs with more than 10 samples are shown**.

We also examined MDR prevalence by genetic lineage among samples with lineage data available. A total of 111 out of 464 (23·9%) samples did not meet the inclusion criteria (containing more than one pneumococcal serotype, or other bacterial species present) and were excluded from this analysis. MDR was identified in 45·6% (n = 161/353) of all samples containing a single pneumococcal serotype. The GPSCs with the largest numbers of MDR isolates were GPSC1 (50/52, 96·2%), GPSC16 (15/16, 93·8%), GPSC13 (44/59, 74·6%), and GPSC23 (18/29, 62·71%) (Fig. 3). MDR in these lineages was closely associated with some serotypes: GPSC1 (19F, 24/50; and 19A, 26/50), GPSC16 (23F, 11/15; and 15B/C, 4/15), GPSC13 (6A, 41/44; and 6B, 3/44), and GPSC23 (all 6B, 18/18).

For the 580 pneumococcal-positive samples collected at 18 (n = 298), 24 (n = 229), and 60 months (n = 53), pneumococcal density was obtained by *lytA* qPCR. Overall pneumococcal density was 5·91 log_10_ GE/ml and was similar between age groups (6·0 log_10_ GE/ml, 5·87 log_10_ GE/ml, and 5·68 log_10_ GE/ml at 18, 24, and 60 months, respectively) (Supplementary Figure S2). We analysed serotype-specific density at 18, 24, and 60 months, focussing on the ten serotypes where carriage was observed in all age groups (6A, 6B, 15A, 15B/C, 19A, 19F, 23A, 23F, 34, 35B) although noting that data are sparse for the age of 60 months. Overall, we found no consistent patterns across age groups. Compared with 18 months, there was some evidence that point estimates for pneumococcal density were lower in children aged 24 months (for serotypes 15B/C and 23A), and 60 months (for serotypes 15A, 15B/C, 23A and 34). In contrast, for serotype 6B we observed some evidence of higher density at ages 24 and 60 months old as shown in Supplementary Figure S3.

## Discussion

Pneumococcal carriage studies in LMICs have primarily focused on populations following PCV introduction. As such, there is limited contemporary data from unvaccinated children. Collecting baseline data in unvaccinated populations is essential for assessing the impact of vaccination in both serotype and lineage distribution. Here, we investigated pneumococcal carriage prevalence, serotype distribution, lineage composition, and AMR prevalence in Vietnamese children. Pneumococcal carriage was 21·7% which is similar to a cluster randomised trial in Nha Trang, Vietnam.^18^ Multiple serotype carriage was low across all ages (1·0%–7·9%), consistent with other findings in Vietnam,^18^ and likely reflects its relatively low overall carriage prevalence. Regional comparisons show that overall carriage prevalence is higher in neighbouring countries: 55.8% in 12–23-month-old children in Laos PDR (with multiple serotype carriage at 8.2%) and 60.1% in the same age group in Mongolia (with multiple serotype carriage at 13.1%).^16^^,^^19^

The serotype distribution across the sampled age groups in Vietnam remained relatively consistent, suggesting that pre-PCV serotype distribution from 6 to 60 months is broadly representative of young children. This consistency can be valuable for public health and future research, as it indicates that sampling unvaccinated children at any age within this range can provide meaningful insights into circulating serotypes. Five of the most common serotypes found in this population were vaccine serotypes (6A, 6B, 19A, 19F, 23F) and the most prevalent non-vaccine serotypes were 15A, 15B/C, and 23A. Serotypes 19F, 23F, and 6B were the most common serotypes found both in carriage and invasive pneumococcal disease cases in South East Asian countries.^20^

We found Synflorix-type carriage was lower compared to Prevenar13-type and Pneumosil-type carriage across all ages. Although Pneumosil is a 10-valent vaccine the prevalence of Pneumosil-types was similar to Prevenar13-types. This is largely explained by the high prevalence of serotype 6A (present in Prevenar13 and Pneumosil, but not in Synflorix) in this population. Interestingly, the carriage prevalence of serotypes within Vaxneuvance and Pneumosil were very similar. While higher-valency vaccines like Vaxneuvance are designed to broaden serotype coverage, their higher costs can limit implementation.^21^ Among the available vaccines, Pneumosil is a lower-cost option, making it a practical choice for many resource-limited settings. Cost-effectiveness studies can be helpful in determining whether higher-valency vaccines offer sufficient benefits to justify their implementation.

Similar to the serotype distribution, lineage distribution was consistent across all ages. The dominant GPSCs identified in Vietnam have also been reported in other countries in the region, including Cambodia and Mongolia, suggesting their widespread circulation across the region.^22^^,^^23^ GPSC1, a globally prevalent pneumococcal lineage typically associated with serotypes 3, 14, 19A, 19F, and 23F, was most frequently linked to serotype 19A in our study, consistent with reports from other Asian countries such as India, Cambodia, China, Thailand, and Malaysia.^24^ GPSC12 was associated with serotype 3 in our study, and is recognised as the sole lineage consistently expressing serotype 3 worldwide.^25^ Globally, GPSC6 has been associated with serotypes 9V and 14, with occasional detection in serotypes 19A, 19F, and 23F.^26^ In our study, GPSC6 was more frequently linked to serotypes 15B/C and 11A, while 9V and 14 were observed less commonly. This divergence from global trends may reflect local selective pressures, which can shape lineage–serotype associations differently across geographic settings highlighting importance of country specific studies. GPSC9, previously reported in Mongolia as predominantly associated with serotype 15A post-vaccine introduction,^22^ was similarly almost entirely composed of serotype 15A in our study.

Antibiotic resistance is a growing concern globally and the burden associated with drug resistant infections in Southeast Asia is high.^27^ In our study, at least one AMR gene was detected in over 98% of pneumococcal samples tested. These results exceed AMR rates reported in other countries using the same DNA microarray methods, including Laos PDR (70%) and Mongolia (82%).^16^^,^^19^ In Vietnam, the use of antibiotics in both human and animal health sectors is high, with Vietnam ranking 11th globally in antibacterial consumption, and with prevalent over-the-counter access to antibiotics, which could also contribute to the low overall carriage prevalence observed.^27^ The most commonly identified AMR genes were *ermB* and *tetM*, conferring resistance to erythromycin (a macrolide) and tetracycline, respectively. Notably, macrolide-resistant pneumococci have been listed on the 2024 WHO Bacterial Priority Pathogens List.^28^ Previous studies have shown that vaccine serotypes are more likely to harbour AMR genes than non-vaccine serotypes,^16^ and our data from Vietnam is consistent with this for *cat* and *mefA*, conferring resistance to chloramphenicol and macrolides, respectively. We also observed higher rates of multi-drug resistance in vaccine serotypes compared with non-vaccine serotypes. Regardless of age, we found a similar pattern of AMR gene prevalence, suggesting that the distribution of these genes is broadly consistent across the ages assessed. The introduction of PCV in Vietnam has potential to reduce pneumococcal AMR. Evidence from other countries with high levels of AMR suggests that PCV vaccination programs may contribute to reducing the prevalence of AMR pneumococci in both children and adults, although the extent of this effect could vary across settings.^11^^,^^29^ In our study, the GPSCs with the highest proportion (>50%) of multidrug-resistant isolates were GPSC1, GPSC16, GPSC13, and GPSC23. These findings are consistent with global trends reported in previous studies. For instance, GPSC1 and GPSC16 have been strongly associated with tetracycline resistance through the presence of the *tetM* gene.^26^ The GPSC23 lineage is globally disseminated; observed in 13 countries across Africa, Asia, Europe, North and South America and it is known to have multi-drug resistance.^30^ GPSC13 is largely associated with serotypes 6A and 6B which are prevalent in Vietnam, however, there is limited information on this lineage in the literature.^31^

This study has several strengths. First, we included older children (60 months), who are often underrepresented in pneumococcal studies. Vietnam has not yet introduced the pneumococcal vaccine into its national immunisation program, providing a valuable opportunity to collect contemporary baseline data that can inform vaccine policy as well as public health strategies both nationally and across the region. Another key strength is the comprehensive sampling of the same children at different ages, providing valuable insights into the pneumococcal carriage during the first five years of life. Additionally, we utilised sensitive molecular techniques to measure pneumococcal density, enabling the detection of multiple serotypes within the same sample and allowing us to infer genetic lineages. These findings provide important data to assess the impact of PCV introduction in Vietnam in the future. However, there are some limitations. Traditional serotyping methods were used for samples collected at 6 and 12 months, and DNA microarray for 18, 24 and 60 months. Importantly, as both techniques rely on an initial culture step, their sensitivity for detecting pneumococci is expected to be comparable. Although molecular serotyping is more sensitive for detecting multiple serotype carriage, the prevalence of multiple carriage in our study was low, making it unlikely that this methodological difference meaningfully influenced our results.^32^^,^^33^ We had a smaller number of children sampled at 60 months, and pneumococcal carriage at that age was low, which could influence the robustness of some of our estimates. In addition, AMR analysis was conducted with data generated using DNA microarray, that includes probes primarily designed for serotype determination, however microarray slides also include oligos for some AMR genes. Our microarray panel cannot reliably distinguish between some allelic variants required for some clinically important resistance mechanisms (penicillin, cephalosporins and fluoroquinolones), therefore the overall resistance burden captured in this study may be underestimated. Our findings should therefore be interpreted as reflecting the ecology of detectable AMR determinants rather than a complete clinical susceptibility profile. In future studies, phenotypic testing and/or whole genome sequencing could be used to provide information on the determinants not detected by DNA microarray.

Our study provides essential baseline data on pneumococcal carriage, including serotype and lineage distribution, as well as antimicrobial resistance, in unvaccinated children. Our findings revealed some of Vietnam's similarities in comparison to neighbouring countries, demonstrating that data from Vietnam are broadly relevant and can help inform vaccine research and policy decisions across the region. Given the ongoing development of newer, higher-valency vaccines, our results establish the importance of making informed vaccine choices that consider local epidemiology. While higher-valency vaccines may expand serotype coverage, they are typically more expensive and could require additional doses. Our results provide valuable data to support vaccine decision-making, particularly in LMICs. Importantly, the consistent trends observed in serotype distribution, lineage, and AMR genes prevalence across age groups suggest that similar epidemiological insights can be obtained from sampling young children at any age, enabling more flexible carriage study designs that can be adapted to local logistical and resource constraints across diverse settings.

## Contributors

FM analysed the data, interpreted the results with input from BT, SM, and CS. FM wrote the first draft of the manuscript with input from BT, SM, and CS. HPT, KB, TLP, DYU, and LTTV were involved in the establishment, day-to-day management, and implementation of VPTI and VPTII trials, generating the data used for this study. For VPTI, TNH was the site principal investigator, while for VPTII, TVN held this role and had overall responsibility for the conduct of the trial in Vietnam. VTTD managed and performed laboratory testing at the Pasteur Institute laboratory for both trials, with contributions from TVP, and HNLT. Acquisition of laboratory data at the Murdoch Children's Research Institute in Melbourne, Australia was done by BO, CP, AWH, LS, for VPTI, VPTII, and VPTII-b; FM helped with acquisition laboratory data for VPTII and VPTII-b. BT performed statistical analyses for both trials, with CDN advising on the statistical plan and analyses for VPTI, and CN advising on sample size and statistical analyses for VPTII. HS-V, and CS oversaw microbiology for the VPTI trial. CS oversaw the microbiology work at MCRI for VPTI, VPTII, managed by BO with input from JH. All data from VPTI, VPTII, and VPTII-b were accessed and verified by BT, clinical data by KB, and laboratory data by VTTD, BO, and JB. KM conceived all studies and had overall responsibility for all aspects of the trials as Principal Investigator. BT and CS accessed and verified the data, with CS taking responsibility for the decision to submit the manuscript. All authors contributed to the refinement and approval of the submitted manuscript.

## Data sharing statement

VPTI and VPTII protocols and informed consent forms have been published previously and are freely available. Data will be made publicly available in accordance with the rules set out by the Gates Foundation. Requests can be made by contacting the corresponding author. Requests must include a relevant proposal detailing the intended use of the data and relevant ethical approval for this proposal, and it requires a signed data sharing agreement.

## Declaration of interests

The authors declare the following financial interests/personal relationships, which may be considered as potential competing interests: KM, CS, and CN were investigators on a clinical research collaboration with Pfizer that examined the effect of PCV vaccination in adults in Mongolia. Additionally, KM, CS, and CN served as investigators on a Merck Investigator Studies Program grant funded by MSD, which studied pneumococcal serotype epidemiology in children with empyema in Australia, with CN contributing as a co-investigator and biostatistician on both projects. CS is an investigator on a pre-study award from Pfizer on the effect of paediatric PCV vaccination in children in Australia. SM has received honoraria from Pfizer for a presentation at a symposium unrelated to this study. BT reports project funding from the Gates Foundation for a PCV trial, paid to their institution. JH is a co-founder and board member of the not-for-profit spin-out company BUGS Bioscience Ltd., and has not received any personal payments. LS has received a salary from MCRI. FM has received support in the form of a stipend from MCRI and the University of Melbourne. The other authors have no relevant conflicts of interest to declare.
