# Supplementary material for: Streptococcus pneumoniae nasopharyngeal carriage in Vietnamese children during the first five years of life: a post hoc analysis
Source: Lancet Reg Health West Pac. 2026 Feb 4;67:101805. doi: 10.1016/j.lanwpc.2026.101805 (PMC12906029; doi:10.1016/j.lanwpc.2026.101805)
Supplement: Supplementary Material [file mmc1.docx]

**APPENDIX**

***Streptococcus pneumoniae* nasopharyngeal carriage in Vietnamese children during the first five years of life: a post hoc analysis**

Table of Contents

[Supplementary Method 1 2](#_Toc216688917)

[1.1 Study site, design, and participants 2](#_Toc216688918)

[1.2 Swabs 2](#_Toc216688919)

[1.3 Study procedures and laboratory analysis 2](#_Toc216688920)

[Supplementary Table S1 - Carriage prevalence of any pneumococci in unvaccinated children from both trials and combined data at each age 2](#_Toc216688921)

[Supplementary Table S2 - Characteristics of VPTI and VPTII study participants 2](#_Toc216688922)

[Supplementary Table S3 - Multiple serotype carriage by age 3](#_Toc216688923)

[Supplementary Table S4 - Frequency of major and minor serotypes among the 73 samples with multiple serotype carriage 3](#_Toc216688924)

[Supplementary Table S5 - Serotype-specific distribution of Global Pneumococcal Sequence Clusters (GPSCs) 4](#_Toc216688926)

[Supplementary Table S6 - AMR in nasopharyngeal samples from unvaccinated Vietnamese children aged 18 months 6](#_Toc216688927)

[Supplementary Table S7 - AMR in nasopharyngeal samples from unvaccinated Vietnamese children aged 24 months 7](#_Toc216688928)

[Supplementary Table S8 - AMR in nasopharyngeal samples from unvaccinated Vietnamese children aged 60 months 8](#_Toc216688929)

[Supplementary Figure 1 - Inferred lineage composition of pneumococcal-positive samples for the six most prevalent serotypes in Vietnam 9](#_Toc216688930)

[Supplementary Figure 2 - Overall pneumococcal density (log_10_ genome equivalents/ml) at different ages in unvaccinated children. 10](#_Toc216688931)

[Supplementary Figure 3 - Serotype-specific density was analysed for serotypes that were detected across all three ages 11](#_Toc216688932)

[References 12](#_Toc216688933)

# **Supplementary Method 1**

**1.1 Study site, design, and participants**

VPTI was carried out in Districts 4 and 7 of Ho Chi Minh City, and VPTII and VPTII-b were carried out in Districts 4, 7, and 8.^1,2^ Districts are subdivided into communes, each featuring a health centre serving as the hub for preventive care. VPTI, VPTII, and VPTII-b took place in one commune health centre per district, with participants recruited from the community within the respective district. Briefly, for the VPTI trial, Vietnamese children (n=1,200) were recruited form the community, at 2 months of age and allocated to one of six different infant PCV schedules, and an additional unvaccinated group (n=199) was recruited at 18 months of age.^1^ For VPTII, Vietnamese children (n=2,500) were recruited at 2 months of age to one of five different PCV schedules.^2^ For VPTII-b, swabs were collected from VPTII participants (n=758) and unvaccinated children from the same community (n=366) at approximately 60 months of age^3^.

## **1.2 Swabs**

VTPI swabs from children aged 2, 6, 9, 12, 18, and 24 months were collected between September 2013 and December 2016; VPTII swabs from children aged 6, 12, 18, and 24 months were collected between June 2017 and June 2019; and VPTII-b swabs from children aged approximately 60 months were collected between September and December 2022.

## **1.3 Study procedures and laboratory analysis**

Nasopharyngeal swabs were collected, stored, and tested according to WHO guidelines.^4^ Swabs collected at 6 months and 12 months were screened for pneumococcus using traditional culture methods by plating on horse blood agar plates containing 5 μg/ml gentamicin followed by assessment of colony morphology, α-haemolysis, and optochin susceptibility then serotyping by latex agglutination and Quellung.^5,6^ Swabs collected at 18, 24, and 60 months were screened for pneumococcus using quantitative real-time polymerase chain reaction (PCR) targeting the *lytA* gene.^7^ Samples that were *lytA* positive (Ct value < 35) or equivocal (Ct value 35-50) were cultured on horse blood agar plates containing 5 μg/ml gentamicin before molecular serotyping, antimicrobial resistance (AMR) gene detection, and lineage inference by DNA microarray using Senti-SP v1.5 microarray (BUGS Bioscience) as described previously.^8,9^ Pneumococci that were non-typeable using conventional sera or were classified as a non-encapsulated genetic lineage by microarray^10,11^, were reported as non-typeable (NT).

**Supplementary Table S1 - Carriage prevalence of any pneumococci in unvaccinated children from both trials and combined data at each age.**

|  | **VPTI** | **VPTII** | **VPTI + VPTII** | **P value*** |
| --- | --- | --- | --- | --- |
| **6m** | 13·5 (26/193) | 16·3 (129/789) | 15·8% (155/982) | 0·745 |
| **12m** | 28·7 (54/188) | 21·4 (163/761) | 22·9% (217/949) | 0·483 |
| **18m** | 26·3 (97/368) | 25·4 (199/782) | 26·5 (305/1150) | 0·635 |
| **24m** | 22·9 (39/170) | 24·9 (189/758) | 24·6 (228/928) | 0·865 |
| **60m** | - | 15·3% (56/366) | 15·3% (56/366) | - |

*The difference between combined data (VPTI + VPTII) and VPTII only was calculated was calculated using Fisher's Exact test. Dashes (-) indicate cases where statistical testing was not performed in cases where both groups were identical

# **Supplementary Table S2 - Characteristics of VPTI and VPTII study participants**

|  | **VPTI** | **VPTII** | **P value** |
| --- | --- | --- | --- |
| **Age, months** |  |  |  |
| 6 | 6·1 (5·0, 6·8) | 6·1 (5·9, 6·6) | 0·083 |
| 12 | 12·1 (12·0, 13.2) | 12·1 (12·0, 12·2) | 0·121 |
| 18 | 18·2 (17·4, 20·3) | 18·0 (17·9, 18·1) | <0·001 |
| 24 | 24·1 (23·4, 26·9) | 24·1 (24·0, 24·3) | 0·334 |
| **Sex** |  |  |  |
| Male | 213/396 (53·8%) | 655/1264 (51·8%) | 0·49 |
| Female | 183/396 (46·2%) | 609/1264 (48·2%) |  |
| **Cigarette smoker in the house** | |  |  |
| Yes | 142/396 (35·85%) | 518/1262* (41·1%) | 0·07 |
| No | 254/396 (64·1%) | 744/1262 (58·9%) |  |
| **District** |  |  |  |
| 4 | 194/396 (48·9%) | 450/1264 (35·6%) | - |
| **7** | 202/396 (51·1%) | 283/1264 (22·4%) | - |
| 8 | - | 531/1264 (42%) | - |

*missing data: cigarette smoker in house, two participants

Dashes (-) indicate cases where statistical testing was not performed as VPTI did not include participants from district 8

**Supplementary Table S3 - Multiple serotype carriage by age. The proportion (95% CI) of pneumococcal-positive swabs in which more than one serotype was detected, excluding and including non-typeable pneumococci**.

|  | **Multiple serotype carriage** | | | |
| --- | --- | --- | --- | --- |
| **Age, months** | **Excluding NT** | | **Including NT** | |
|  | **% (n/N)** | **95%CI** | **% (n/N)** | **95%CI** |
| **6** | 1·3 (2/155) | 0·1-4·5 | 1·9% (3/155) | 0·4-5·5 |
| **12** | 0·9 (2/217) | 0·1-3·2 | 2·3% (5/217) | 0·7-5·3 |
| **18** | 3·7 (11/296) | 1·8-6·5 | 12·8% (38/296) | 9·2-17·2 |
| **24** | 6·6 (15/228) | 3·7-10·6 | 10·1% (23/228) | 6·5-14·7 |
| **60** | 3·6 (2/56) | 0·4-12·9 | 7·4% (4/56) | 1·9-17·2 |

# **Supplementary Table S4 – Frequency of major and minor serotypes among the 73 samples with multiple serotype carriage. Data are shown as % (n/N), where n is the number of samples containing the given serotype and N is the total number of samples within the respective category.**

|  | **Major** | **Minor** |
| --- | --- | --- |
| **Serotype** | **% (n/N)** | **% (n/N)*** |
| **4** | 0% (0/73) | 1.3% (1/77) |
| **9V** | 1.3% (1/73) | 1.3% (1/77) |
| **34** | 2.7% (2/73) | 2.6% (2/77) |
| **14** | 4.1% (3/73) | 6.5% (5/77) |
| **23A** | 5.4% (4/73) | 5.2% (4/77) |
| **15B/C** | 5.4% (4/73) | 2.6% (2/77) |
| **19A** | 4.1% (3/73) | 1.3% (1/77) |
| **15A** | 4.1% (3/73) | 3.9% (3/77) |
| **19F** | 12.3% (9/73) | 13% (10/77) |
| **6B** | 10.9% (8/73) | 6.5% (5/77) |
| **23F** | 15.1% (11/73) | 1.3% (1/77) |
| **6A** | 13.7% (10/73) | 14.3% (11/77) |
| **13** | 2.7% (2/73) | 0% (0/77) |
| **35B** | 1.3% (1/73) | 0% (0/77) |
| **NT** | 16.4% (12/73) | 40.2% (31/77) |

* The total N for minor serotypes exceeds 73 because four samples carried three serotypes.

**Supplementary Table S5 - Serotype-specific distribution of Global Pneumococcal Sequence Clusters (GPSCs). For each serotype, the table shows the number and percentage of samples assigned to each GPSC, presented as n/N (%), where n is the number of samples belonging to a given GPSC and N is the total number of samples for that serotype.**

| **Serotype** | **GPSC** | **n/N (%)** |
| --- | --- | --- |
| **11A** | 6 | 9/9 (100%) |
| **13** | 134 | 2/3 (66·7%) |
|  | 230 | 1/3 (33·3%) |
| **14** | 9 | 6/15 (40%) |
|  | 18 | 4/15 (26·7%) |
|  | 1 | 2/15 (13·3%) |
|  | 279 | 2/15 (13·3%) |
|  | 6 | 1/15 (6·7%) |
| **15A** | 9 | 34/34 (100%) |
| **15B/C** | 6 | 19/30 (63·3%) |
|  | 16 | 6/30 (20%) |
|  | 48 | 5/30 (16·7%) |
| **18C** | 142 | 1/1(100%) |
| **19A** | 1 | 31/36 (86·1%) |
|  | 10 | 4/36 (11·1%) |
|  | 9 | 1/36 (2·8%) |
| **19F** | 1 | 38/38 (100%) |
| **23A** | 5 | 19/21 (90·5%) |
|  | 10 | 1/21 (4·8%) |
|  | 6 | 1/21 (4·8%) |
| **23F** | 14 | 36/54 (66·7%) |
|  | 16 | 15/54 (27·8%) |
|  | 624 | 2/54 (3·7%) |
|  | 9 | 1/54 (1·9%) |
| **3** | 12 | 3/3 (100%) |
| **34** | 45 | 14/14 (100%) |
| **35A** | 43 | 1/1 (100%) |
| **35B** | 59 | 3/3 (100%) |
| **6A** | 13 | 73/74 (98·6%) |
|  | 623 | 1/74 (1·4%) |
| **6B** | 23 | 40/48 (83·3%) |
|  | 13 | 3/48 (6·2%) |
|  | 47 | 3/48 (6·2%) |
|  | 321 | 1/48 (2·1%) |
|  | 852 | 1/48 (2·1%) |
| **6C** | 47 | 1/1 (100%) |
| **9V** | 6 | 2/2 (100%) |
| **NT2** | 397 | 55/62 (88·7%) |
|  | 324 | 4/62 (6·5%) |
|  | 43 | 1/62 (1·6%) |
|  | 628 | 1/62 (1·6%) |
|  | 777 | 1/62 (1·6%) |
| **NT3b** | 60 | 5/5 (100%) |
| **NT4a** | 397 | 1/2 (50%) |
|  | 806 | 1/2 (50%) |
| **NT4b** | 397 | 2/3 (66·7%) |
|  | 60 | 1/3 (33·3%) |

GPSC, Global Pneumococcal Sequence Cluster. NT2, NT3b, NT4a, NT4b refer to different lineages of non-encapsulated pneumococci

**Supplementary Table S6 - AMR in nasopharyngeal samples from unvaccinated Vietnamese children aged 18 months. The detection of AMR genes was shown for Synflorix-types, and Non-Synflorix- types; Pneumosil-types and Non-Pneumosil-types.**

| **18 months old** | | | | | | | |
| --- | --- | --- | --- | --- | --- | --- | --- |
| **AMR gene** | **Encodes resistance to** | **Synflorix-type (N=82)** | **Non-Synflorix-type (N=108)** | **P value*** | **Pneumosil-type (N=143)** | **Non-Pneumosil-type (N=59)** | **P value*** |
| *aphA3* | Kanamycin | 2 (2%) | 1 (1%) | 0·397 | 3 (2%) | 0 (0%) | 0·353 |
| *cat* | Chloramphenicol | 18 (22%) | 25 (23%) | 0·494 | 47 (33%) | 3 (5%) | <0·001 |
| *ermB* | Erythromycin | 69 (84%) | 103 (95%) | 0·009 | 131 (92%) | 53 (90%) | 0·436 |
| *ermC* | Erythromycin | 6 (7%) | 6 (6%) | 0·419 | 9 (6%) | 3 (5%) | 0·515 |
| *mefA* | Macrolides | 29 (35%) | 18 (17%) | 0·003 | 50 (35%) | 3 (5%) | <0·001 |
| *tetK* | Tetracycline | 5 (6%) | 2 (2%) | 0·126 | 5 (3%) | 2 (3%) | 0·666 |
| *tetL* | Tetracycline | 0 (0%) | 0 (0%) | - | 0 (0%) | 0 (0%) | - |
| *tetM* | Tetracycline | 71 (87%) | 105 (97%) | 0·006 | 132 (92%) | 56 (95%) | 0·373 |
| *tetO* | Tetracycline | 0 (0%) | 0 (0%) | - | 0 (0%) | 0 (0%) | - |
| *sat4* | Streptothricin | 2 (2%) | 1 (1%) | 0·397 | 3 (2%) | 0 (0%) | 0·353 |
| any |  | 80 (98%) | 106 (98%) | 0·580 | 141 (99%) | 57 (97%) | 0·455 |
| Multi-drug resistance** |  | 43 (52%) | 56 (44%) | 0·092 | 91 (64%) | 7 (12%) | <0·001 |

AMR, Antimicrobial resistance genes

*The difference between Synflorix-type vs non-Synflorix-types, Pneumosil-types vs non-Pneumosil-types was calculated using Fisher’s exact test

**Multi-drug resistance is defined as the presence of three or more AMR genes

AMR and their associated resistance: *aphA3* (kanamycin), *cat* (chloramphenicol), *ermB, ermC* (erythromycin), *mefA* (macrolides), *tetK, tetM, tetO, tetL* (tetracycline), *sat4* (streptothricin). The analysis was restricted to samples containing a single pneumococcal type with no other species present. Dashes (-) indicate cases where statistical testing was not performed due to the absence of the respective AMR genes in both groups, or in cases where both groups were identical.

**Supplementary Table S7 - AMR in nasopharyngeal samples from unvaccinated Vietnamese children aged 24 months. The detection of AMR genes was shown for Synflorix-types, and Non-Synflorix- types; Pneumosil-types and Non-Pneumosil-types.**

| **24 months old** | | | | | | | |
| --- | --- | --- | --- | --- | --- | --- | --- |
| **AMR gene** | **Encodes resistance to** | **Synflorix-type (N=22)** | **Non-Synflorix-type (N=43)** | **P value*** | **Pneumosil-type (N=37)** | **Non-Pneumosil-type (N=30)** | **P value*** |
| *aphA3* | Kanamycin | 2 (9%) | 1 (2%) | 0·263 | 3 (8%) | 1 (3%) | 0·390 |
| *cat* | Chloramphenicol | 11 (50%) | 10 (23%) | 0.030 | 18 (49%) | 3 (10%) | 0·001 |
| *ermB* | Erythromycin | 22 (100%) | 42 (98%) | 0.662 | 37 (100%) | 29 (97%) | 0·448 |
| *ermC* | Erythromycin | 3 (14%) | 5 (12%) | 0.552 | 6 (16%) | 3 (10%) | 0·355 |
| *mefA* | Macrolides | 6 (27%) | 5 (12%) | 0.109 | 11 (30%) | 1 (3%) | 0·005 |
| *tetK* | Tetracycline | 2 (9%) | 1 (2%) | 0.263 | 3 (8%) | 1 (3%) | 0·39 |
| *tetL* | Tetracycline | 0 (0%) | 0 (0%) | - | 0 (0%) | 0 (0%) | - |
| *tetM* | Tetracycline | 22 (100%) | 42 (98%) | 0.662 | 37 (100%) | 29 (97%) | 0·448 |
| *tetO* | Tetracycline | 0 (0%) | 1 (2%) | 0.662 | 0 (0%) | 1 (3%) | 0·448 |
| *sat4* | Streptothricin | 2 (9%) | 0 (0%) | 0.111 | 3 (8%) | 0 (0%) | 0·162 |
| any |  | 22 (100%) | 43 (100%) | - | 37 (100%) | 30 (100%) | - |
| Multi-drug resistance** |  | 18 (82%) | 19 (44%) | 0.004 | 30 (81%) | 10 (29%) | <0·001 |

AMR, Antimicrobial resistance genes

*The difference between Synflorix-type vs non-Synflorix-types, Pneumosil-types vs non-Pneumosil-types was calculated using Fisher’s exact test

**Multi-drug resistance is defined as the presence of three or more AMR genes

AMR and their associated resistance: *aphA3* (kanamycin), *cat* (chloramphenicol), *ermB, ermC* (erythromycin), *mefA* (macrolides), *tetK, tetM, tetO, tetL* (tetracycline), *sat4* (streptothricin). The analysis was restricted to samples containing a single pneumococcal type with no other species present. Dashes (-) indicate cases where statistical testing was not performed due to the absence of the respective AMR genes in both groups, or in cases where both groups were identical.

**Supplementary Table S8 - AMR in nasopharyngeal samples from unvaccinated Vietnamese children aged 60 months. The detection of AMR genes was shown for Synflorix-types, and Non-Synflorix- types; Pneumosil-types and Non-Pneumosil-types.**

| **60 months old** | | | | | | | |
| --- | --- | --- | --- | --- | --- | --- | --- |
| **AMR gene** | **Encodes resistance to** | **Synflorix-type (N=11)** | **Non-Synflorix-type (N=25)** | **P value*** | **Pneumosil-type (N=22)** | **Non-Pneumosil-type (N=14)** | **P value*** |
| *aphA3* | Kanamycin | 0 (0%) | 0 (0%) | - | 0 (0%) | 0 (0%) | - |
| *cat* | Chloramphenicol | 1 (9%) | 6 (24%) | 0·291 | 5 (23%) | 2 (14%) | 0·433 |
| *ermB* | Erythromycin | 9 (82%) | 25 (100%) | 0·087 | 20 (91%) | 14 (100%) | 0·367 |
| *ermC* | Erythromycin | 1 (9%) | 1 (4%) | 0·524 | 2 (9%) | 0 (0%) | 0·367 |
| *mefA* | Macrolides | 3 (27%) | 3 (12%) | 0·252 | 6 (27%) | 0 (0%) | 0·038 |
| *tetK* | Tetracycline | 0 (0%) | 1 (4%) | 0·694 | 0 (0%) | 1 (7%) | 0·389 |
| *tetL* | Tetracycline | 0 (0%) | 0 (0%) | - | 0 (0%) | 0 (0%) | - |
| *tetM* | Tetracycline | 9 (82%) | 25 (100%) | 0·087 | 20 (91%) | 14 (100%) | 0·367 |
| *tetO* | Tetracycline | 0 (0%) | 0 (0%) | - | 0 (0%) | 0 (0%) | - |
| *sat4* | Streptothricin | 0 (0%) | 0 (0%) | - | 0 (0%) | 0 (0%) | - |
| any |  | 11 (100%) | 25 (100%) | - | 22 (100%) | 14 (100%) | - |
| Multi-drug resistance** |  | 3 (27%) | 11 (44%) | 0·285 | 11 (50%) | 3 (21%) | 0·085 |

AMR, Antimicrobial resistance genes

*The difference between Synflorix-type vs non-Synflorix-types, Pneumosil-types vs non-Pneumosil-types was calculated using Fisher’s exact test

**Multi-drug resistance is defined as the presence of three or more AMR genes

AMR and their associated resistance: *aphA3* (kanamycin), *cat* (chloramphenicol), *ermB, ermC* (erythromycin), *mefA* (macrolides), *tetK, tetM, tetO, tetL* (tetracycline), *sat4* (streptothricin). The analysis was restricted to samples containing a single pneumococcal type with no other species present. Dashes (-) indicate cases where statistical testing was not performed due to the absence of the respective AMR genes in both groups, or in cases where both groups were identical.


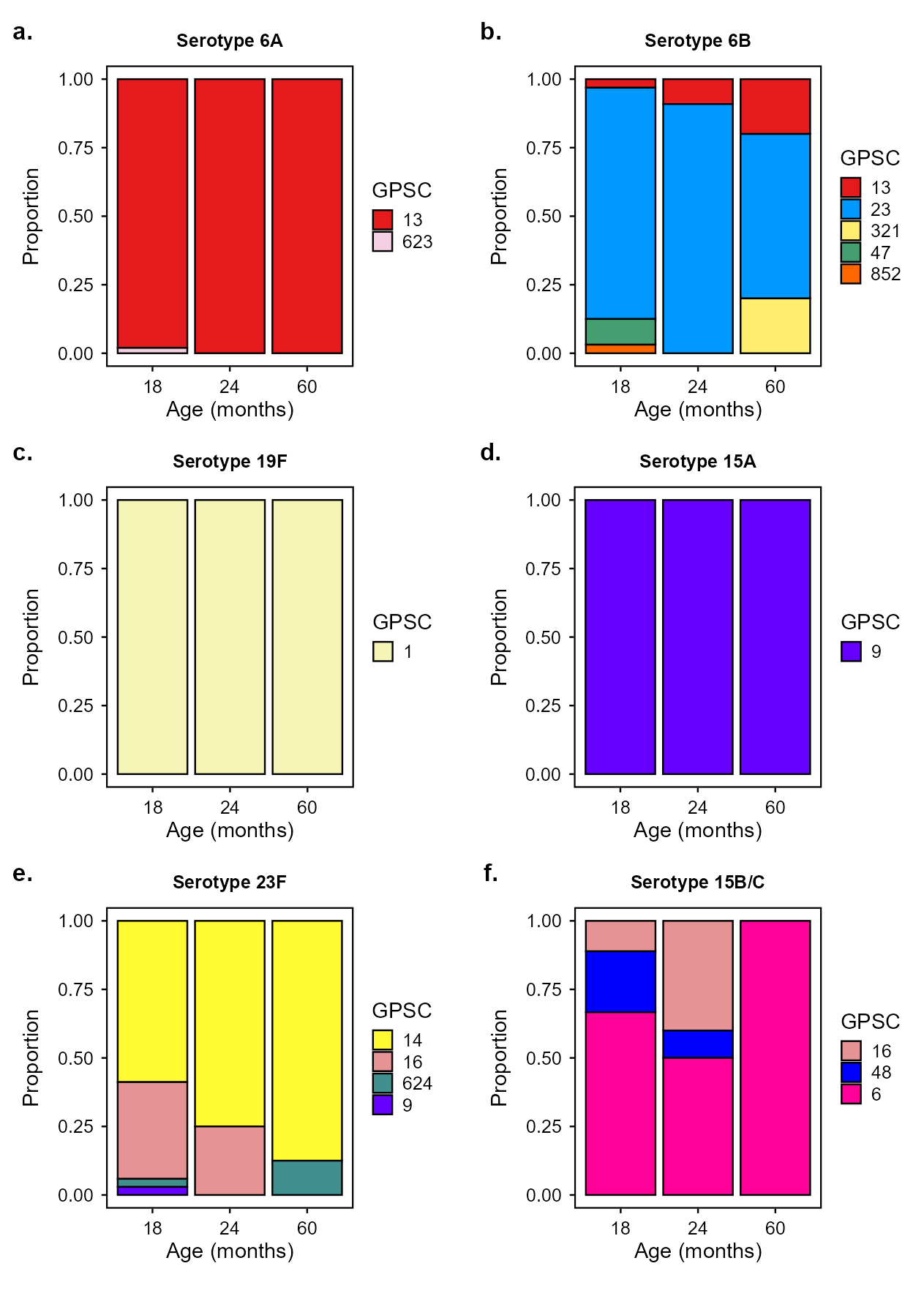


Supplementary Figure 1 - Inferred lineage composition of pneumococcal-positive samples for the six most prevalent serotypes in Vietnam 6A, 6B, 19F, 15A, 23F and 15B/C at 18, 24 and 60 months (shown in panels a to f). The serotype was the ‘major’ serotype detected by microarray. Lineages were inferred for the calls with the highest relative abundance using DNA microarray and were analysed for lineage composition. Bars are coloured by lineage. GPSC, Global Pneumococcal Sequence Cluster.


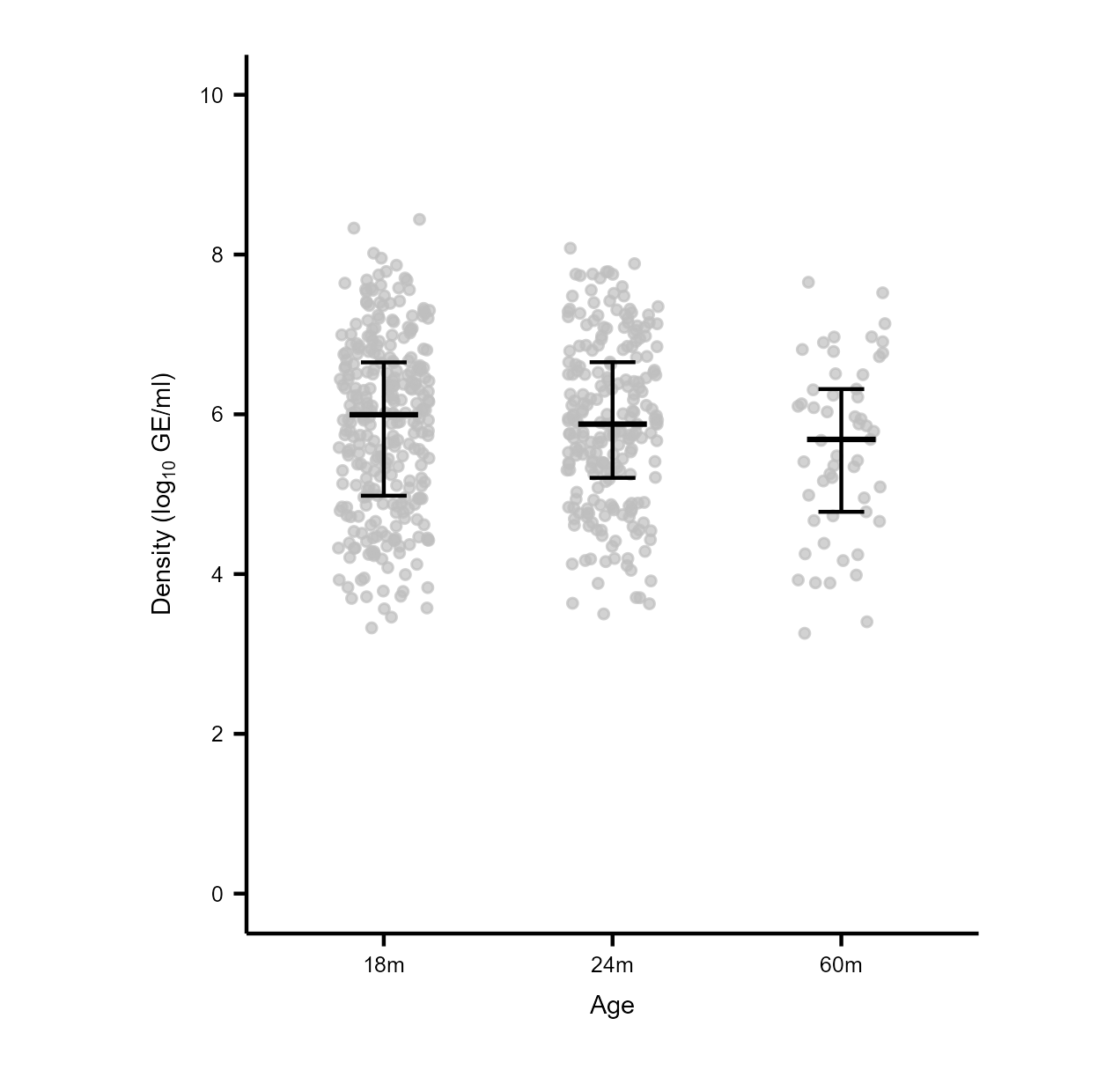


Supplementary Figure 2 – Overall pneumococcal density (log_10_ genome equivalents/ml) at different ages in unvaccinated children. Bars indicate the median (IQR). Statistical significance between each age was determined using the Mann-Whitney U test. For all comparisons p-values > 0·05.


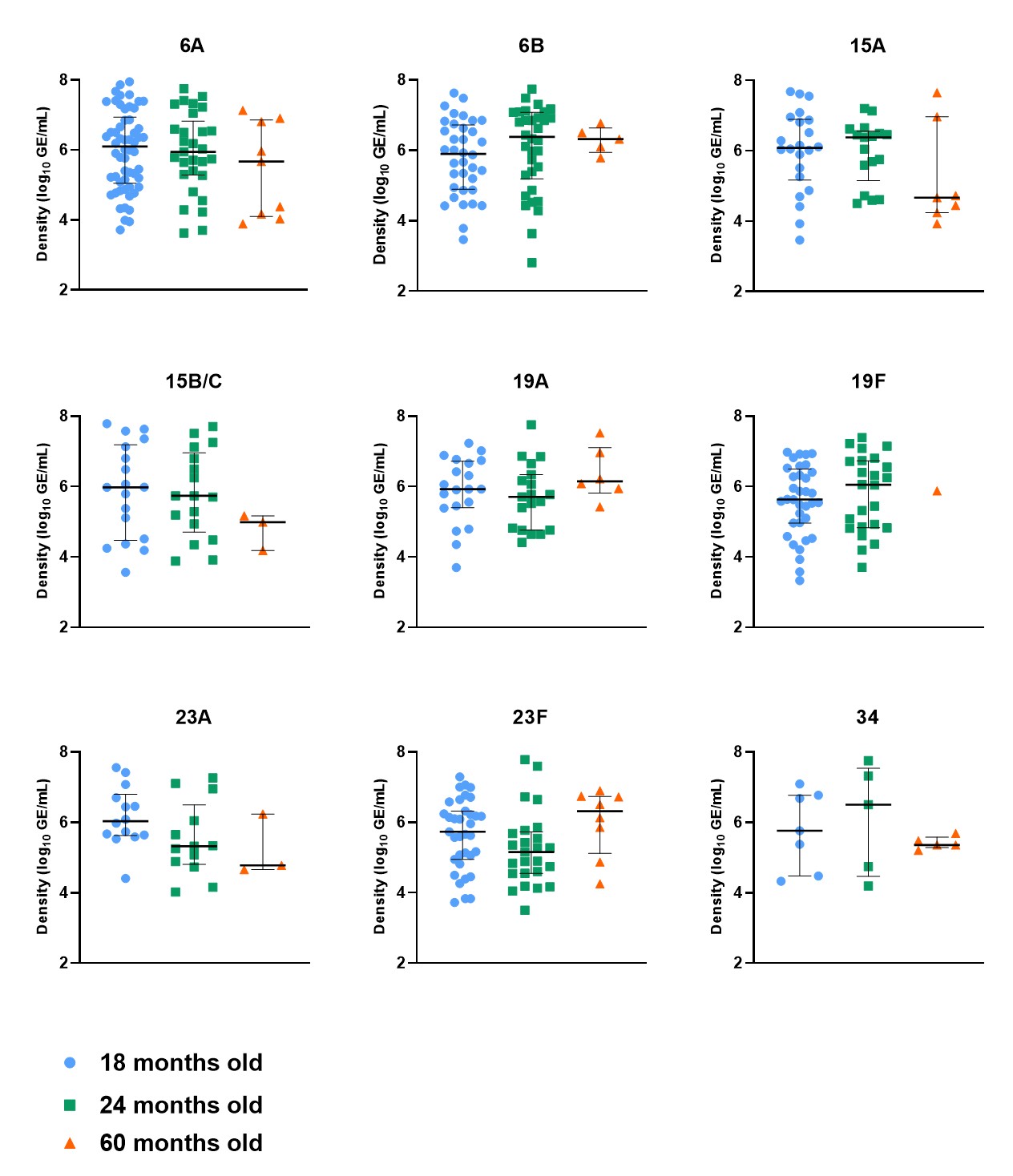


Supplementary Figure 3 - Serotype-specific density was analysed for serotypes that were detected across all three ages (18, 24, and 60-month-old children). Data are presented as medians with interquartile ranges (IQRs). For each serotype, the differences in density between ages (18 months vs. 24 months, and 24 months vs. 60 months); were compared using a Mann–Whitney U test; For all serotypes all comparisons shown p-values > 0·05. Data not shown for serotype 35B (only one positive case detected at each time point).

# **References**

1. Temple B, Toan NT, Uyen DY, et al. Evaluation of different infant vaccination schedules incorporating pneumococcal vaccination (The Vietnam Pneumococcal Project): protocol of a randomised controlled trial. *BMJ Open* 2018; **8**(6): e019795.

2. Temple B, Tran HP, Dai VTT, et al. Simplified 0+1 and 1+1 pneumococcal vaccine schedules in Ho Chi Minh City, Vietnam: protocol for a randomised controlled trial. *BMJ Open* 2021; **11**(11): e056505.

3. Tran HP. Pneumococcal carriage among 4 to 5 year-old children with different pneumococcal vaccination history in Ho Chi Minh City, Vietnam. International Symposium on Pneumococci and Pneumococcal Diseases (ISPPD). Cape Town, South Africa; 2024.

4. Satzke C, Turner P, Virolainen-Julkunen A, et al. Standard method for detecting upper respiratory carriage of *Streptococcus pneumoniae*: updated recommendations from the World Health Organization Pneumococcal Carriage Working Group. *Vaccine* 2013; **32**(1): 165-79.

5. Ortika BD, Habib M, Dunne EM, Porter BD, Satzke C. Production of latex agglutination reagents for pneumococcal serotyping. *BMC Res Notes* 2013; **6**: 49.

6. Habib M, Porter BD, Satzke C. Capsular serotyping of *Streptococcus pneumoniae* using the Quellung reaction. *J Vis Exp* 2014; (84): e51208.

7. Carvalho Mda G, Tondella ML, McCaustland K, et al. Evaluation and improvement of real-time PCR assays targeting lytA, ply, and psaA genes for detection of pneumococcal DNA. *J Clin Microbiol* 2007; **45**(8): 2460-6.

8. Dunne EM, Satzke C, Ratu FT, et al. Effect of ten-valent pneumococcal conjugate vaccine introduction on pneumococcal carriage in Fiji: results from four annual cross-sectional carriage surveys. *Lancet Glob Health* 2018; **6**(12): e1375-e85.

9. Satzke C, Dunne EM, Porter BD, Klugman KP, Mulholland EK, PneuCarriage project g. The PneuCarriage Project: A Multi-Centre Comparative Study to Identify the Best Serotyping Methods for Examining Pneumococcal Carriage in Vaccine Evaluation Studies. *PLoS Med* 2015; **12**(11): e1001903; discussion e.

10. Newton R, Hinds J, Wernisch L. Empirical Bayesian models for analysing molecular serotyping microarrays. *BMC Bioinformatics* 2011; **12**: 88.

11. Turner P, Hinds J, Turner C, et al. Improved detection of nasopharyngeal cocolonization by multiple pneumococcal serotypes by use of latex agglutination or molecular serotyping by microarray. *J Clin Microbiol* 2011; **49**(5): 1784-9.
